# A multi-task CNN learning model for taxonomic assignment of human viruses

**DOI:** 10.1186/s12859-021-04084-w

**Published:** 2021-06-02

**Authors:** Haoran Ma, Tin Wee Tan, Kenneth Hon Kim Ban

**Affiliations:** 1 Department of Biochemistry, Yong Loo Lin School of Medicine, National University of Singapore, 117592 Singapore, Singapore; 2 National Supercomputing Centre (NSCC), 138632 Singapore, Singapore

**Keywords:** Convolutional neural network, Deep learning, Taxonomic assignment, Genomic coverage, Naïve Bayesian network

## Abstract

**Background:**

Taxonomic assignment is a key step in the identification of human viral pathogens. Current tools for taxonomic assignment from sequencing reads based on alignment or alignment-free *k*-mer approaches may not perform optimally in cases where the sequences diverge significantly from the reference sequences. Furthermore, many tools may not incorporate the genomic coverage of assigned reads as part of overall likelihood of a correct taxonomic assignment for a sample.

**Results:**

In this paper, we describe the development of a pipeline that incorporates a multi-task learning model based on convolutional neural network (MT-CNN) and a Bayesian ranking approach to identify and rank the most likely human virus from sequence reads. For taxonomic assignment of reads, the MT-CNN model outperformed Kraken 2, Centrifuge, and Bowtie 2 on reads generated from simulated divergent HIV-1 genomes and was more sensitive in identifying SARS as the closest relation in four RNA sequencing datasets for SARS-CoV-2 virus. For genomic region assignment of assigned reads, the MT-CNN model performed competitively compared with Bowtie 2 and the region assignments were used for estimation of genomic coverage that was incorporated into a naïve Bayesian network together with the proportion of taxonomic assignments to rank the likelihood of candidate human viruses from sequence data.

**Conclusions:**

We have developed a pipeline that combines a novel MT-CNN model that is able to identify viruses with divergent sequences together with assignment of the genomic region, with a Bayesian approach to ranking of taxonomic assignments by taking into account both the number of assigned reads and genomic coverage. The pipeline is available at GitHub via https://github.com/MaHaoran627/CNN_Virus.

**Supplementary Information:**

The online version contains supplementary material available at 10.1186/s12859-021-04084-w.

## Background

In recent years, viral diseases have emerged or re-emerged, such as SARS (Severe Acute Respiratory Syndrome) in 2003–2004, and Ebola in 2014, 2017, and 2018. Most recently, the emergence of COVID-19 in November 2019 caused by SARS-CoV-2 has impacted human society profoundly, highlighting the need to rapid identification of emergent pathogenic viruses that may be divergent from previously known viruses. Identification of a likely pathogenic virus from patient samples, most commonly from short-read sequences, relies on the number of taxonomically-assigned reads as well as the coverage of reads across the closest reference genome.

Taxonomic assignment, which aims to assign the correct taxonomic label to an unlabeled sequencing read based on reference databases, is a key step in identifying human viruses. Current taxonomic tools, including sequence alignment-based tools such as Bowtie [1] and *k*-mer searching-based tools such as Kraken [2]. However, these tools may not perform optimally in cases where the sequences diverge significantly from the reference sequences, such as viruses with high mutation rates. For alignment-based tools, the accuracy of alignment can be influenced by the error rates of the sequencing data [3]. For *k*-mer based tools, they may not be able to deal with mutated *k*-mers that not in the reference database. In addition, sensitivity and specificity are dependent of the *k*-mer length; a large *k* value can lead to lower sensitivity, while a small *k* value can lead to non-specific matches to the reference database [4]. Therefore, there is a need to develop new approaches that are robust enough to identify reads from divergent viruses with high mutation rates.

**Fig. 1:**
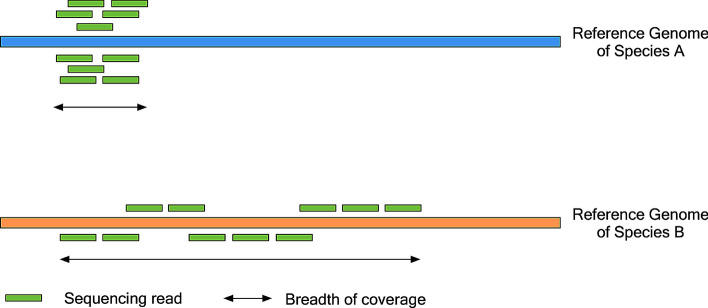
Incorporating genomic coverage information in taxonomic assignment. In this example, viruses A and B are two candidate pathogens in a taxonomic report with the same number of assigned sequencing reads. As the genomic coverage on virus B is broader than virus A, virus B could be considered as having a higher likelihood of a correct assignment in the taxonomic report

In addition to challenges in taxonomic assignment of reads for divergent viruses, taxonomic assignment tools may not incorporate sequence coverage of assigned reads that could help indicate the likelihood of a correct taxonomic assignment of reads for the overall sample. For example, in a taxonomic report of Centrifuge [5], identified pathogens are ranked based on their taxonomic IDs. In Kraken 2 [6], identified pathogens are displayed in a taxonomic tree structure and ranked based on the percentage of identified reads. The interpretability of a taxonomic report could be improved by considering the percentage of the reference genome covered by assigned reads [7]. This breadth of coverage reflects the overall distribution of assigned reads, which may aid in ranking the likelihood of taxonomic assignments. In the example shown in Fig. 1, both viruses A and B have the same number of assigned sequencing reads in a taxonomic report. However, as the coverage of reads on virus B is broader than virus A, virus B could be ranked as having a higher likelihood of being the correctly identified viral species.

To address some of these challenges, deep learning techniques such as convolutional neural networks (CNN) can be adopted. CNNs can be used to learn features from sequential data such images using convolutional kernels that function as feature detectors. Because of its ability of learning generalizable features from sequence data, CNNs may harnessed for improving the taxonomic assignment of viral species that have diverged from reference genomes. Recently, several deep learning approaches have been used for classifying viruses from metagenomic reads [8, 9] and a tree-structure CNN pipeline [10] has been developed for virus taxonomic classification. However, despite the progress in adapting deep learning models for viral taxonomic assignment, the architectures may not be sufficient for handling highly divergent viral species given the shallow depth of the networks. Furthermore, none of these deep learning models are able to estimate the genomic position of reads required for deriving the genomic coverage of assigned reads that may be used for ranking the likelihood of taxonomic assignments in samples.

In this paper, we sought to address the challenges of taxonomic identification of divergent human viruses and the utilization of genome coverage information for ranking of taxonomic assignments to improve the interpretability of taxonomic reports. We describe the development of a pipeline that incorporates a multi-task CNN model (MT-CNN) that can perform taxonomic assignment and estimation of genomic region for assigned reads for human viruses, together with a naïve Bayesian network that considers both the taxonomic assignments and the genomic coverage for the ranking of likely human viruses from sequence data. We demonstrate that our MT-CNN model is able to identify divergent human viruses and to estimate the genomic regions of assigned reads, and show that the Bayesian network is able to re-rank the likelihood of taxonomic assignment by incorporating information about the genomic coverage of assigned reads.

## Implementation

### Overview of pipeline

**Fig. 2:**
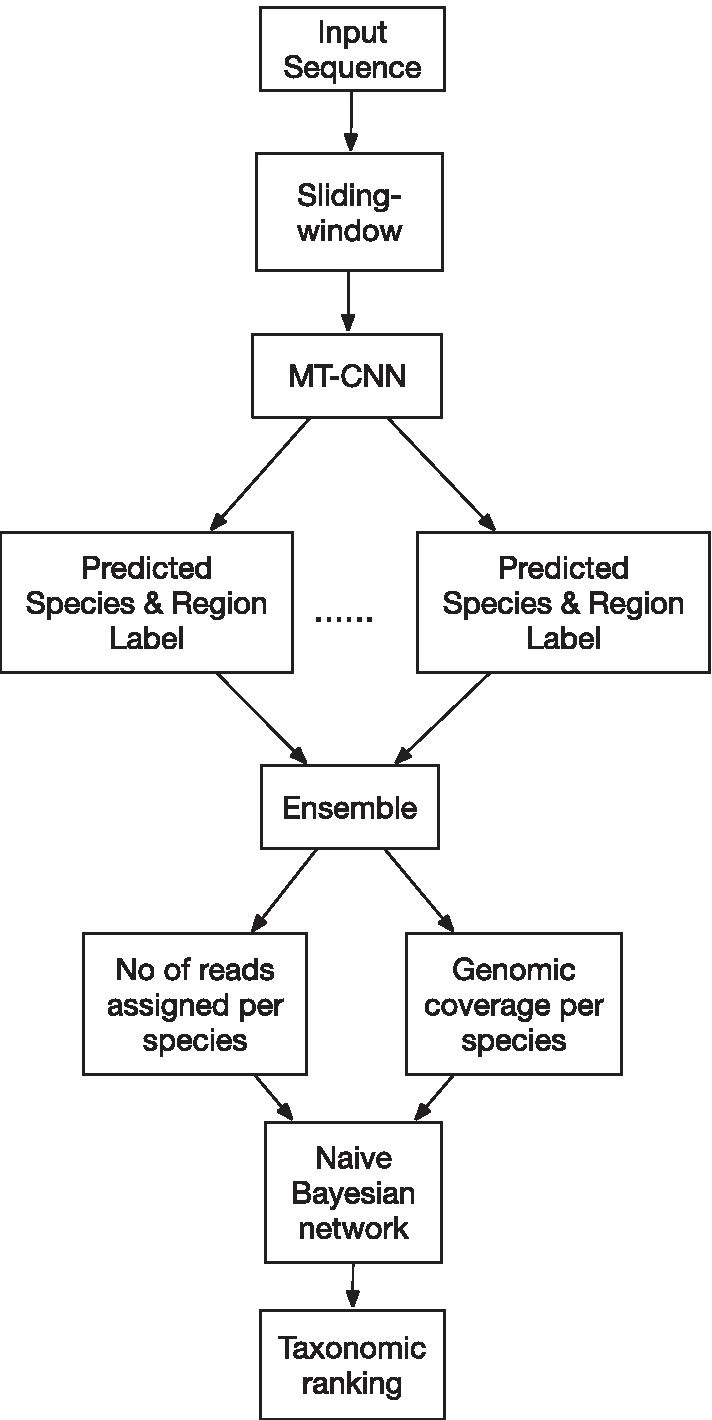
Overview of the MT-CNN/Bayesian pipeline. The pipeline incorporates a sliding window for splitting input sequences into 50-mers for prediction by the MT-CNN model. The outputs of the model are ensembled to identify the number of read and genomic coverage per species in the sample. Finally, both parameters are used to estimate a taxonomic ranking using a naïve Bayes network

The pipeline for taxonomic assignment and ranking (Fig. 2) was implemented in several parts: (1) a multi-task CNN (MT-CNN) model for taxonomic assignment and estimation of the genomic region of 50-mer sequences, (2) a sliding window and ensembling algorithm for handling variable length sequences ≥ 50 bp for the MT-CNN model, and (3) a naïve Bayes network for taxonomic ranking based on number of assigned reads and genomic coverage derived from the MT-CNN model.

### Development of the multi-task CNN (MT-CNN) model

**Fig. 3:**
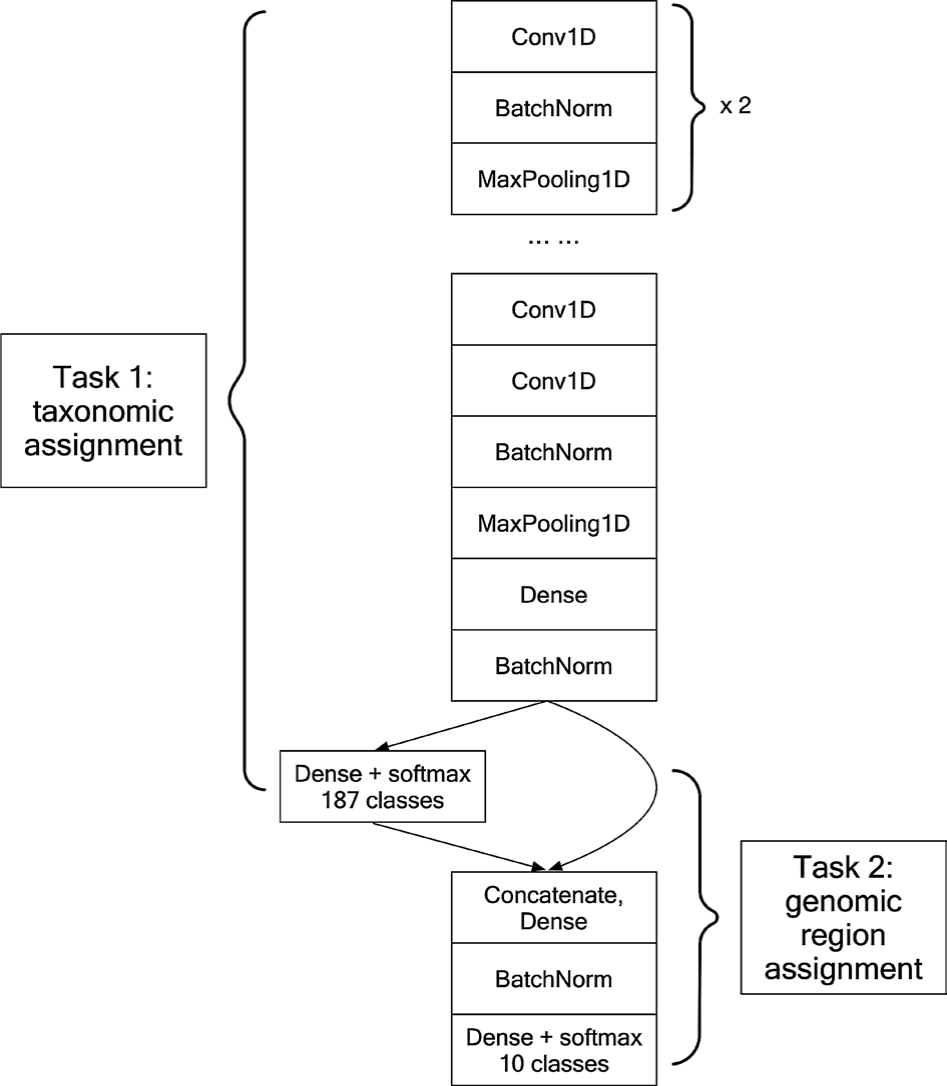
The architecture of the multi-task CNN learning model. In this multi-task model, the CNN network data performs the first task of taxonomic assignment. The final layer of the CNN model is concatenated with the predicted species label as input to a fully connected model for the second task of assigning the genomic region for the assigned taxonomic label

A multi-task CNN (MT-CNN) model was developed to perform the taxonomic assignment and the estimation of the genomic region of 50-mer sequences (Fig. 3). For the taxonomic assignment task, the model was based on VGGNet (VGG-11) [11], initially developed for image classification but modified to process input DNA sequences that were one-hot encoded as A, T, C, G. Modifications included varying the number of convolutional layers, the number of neurons in each layer, altering the frame size of convolutional kernels, and converting the convolutional and max-pooling layers from 2 to 1D.

For the second task of estimating the genomic position of a sequence, the model was extended to predict the region of the sequence in a genome (out of 10 equipartitioned regions) that was taxonomically assigned by the first task. To implement this, the feature map from the last dense layer of the first taxonomic assignment task was concatenated with the predicted species labels used as the input for the second task, analogous to transfer learning [12]. For this task, a fully connected model was used in the classification of genomic regions.

In the final model, several batch normalization layers were included for improving the classification accuracy and accelerating the training process [13]. The activation function in each layer was ReLU [14], except for the two dense layers for prediction using Softmax [15].

For training, the cross-entropy loss function for both classification tasks was used and the sum of their losses was defined as the total loss of the model. Training was performed using the Adam optimizer [16] and hyper-parameter tuning was done using the Talos framework (https://github.com/autonomio/talos). The final MT-CNN model was implemented using Keras 2.2.4 (https://keras.io/) in Python 3.7 (https://www.python.org).

### Sliding window strategy for predictions of sequences longer that 50 bases

**Fig. 4:**
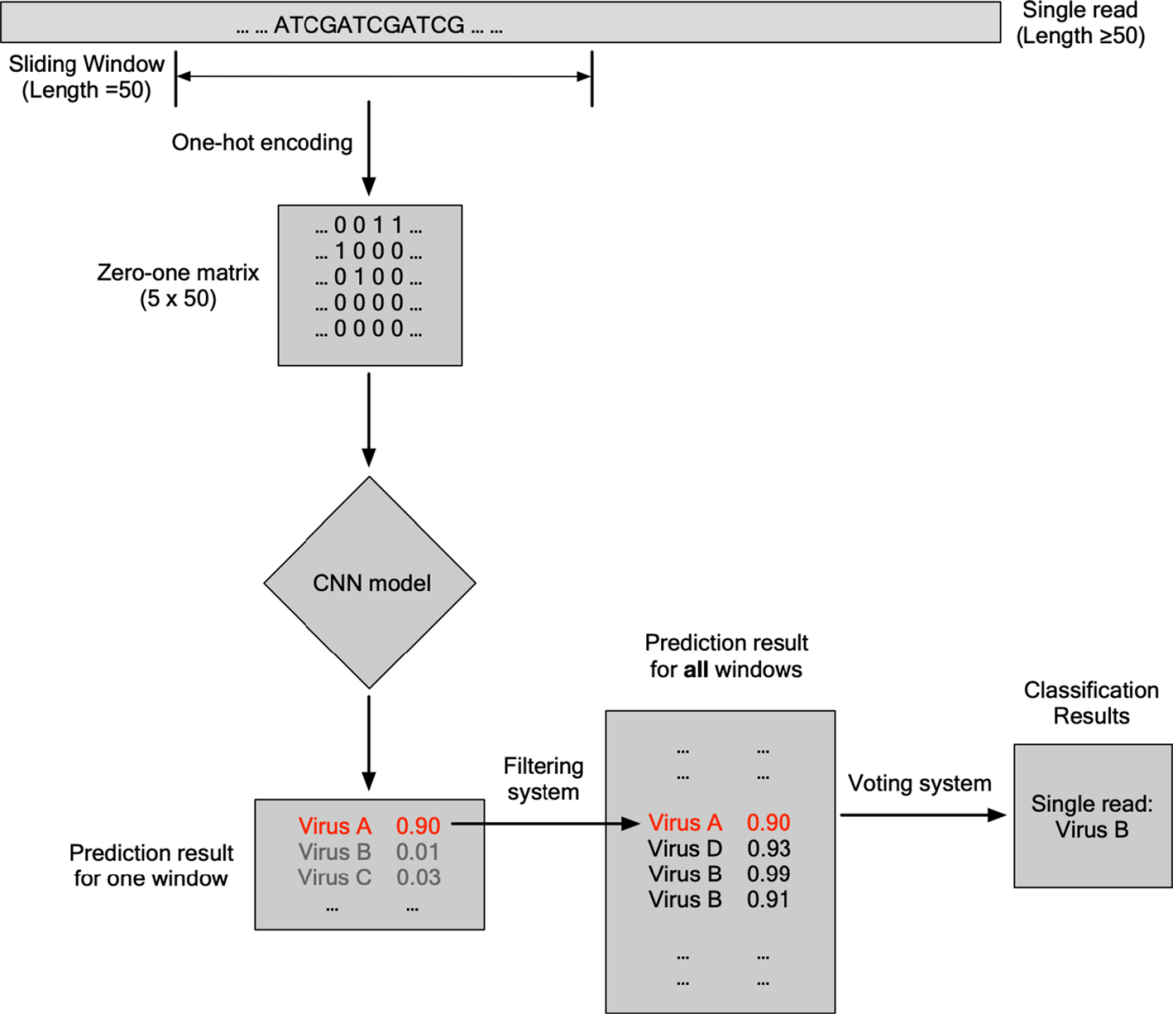
The sliding window and ensembling strategy. For input reads that are longer than 50 bp, the read is split into overlapping 50-mers using a 1-bp sliding window. The predictions of each 50-mer sequences will be ensembled by a hard-voting system with a pre-defined threshold for the final predicted labels

To extend the model’s ability to perform predictions on longer sequences (typically 150–300 for current short-read sequences), a sliding-window strategy was implemented (Fig. 4).

Briefly, input reads with length N (N ≥ 50) were split into (N – 49) overlapping 50-mers with a 1-bp sliding window. Each 50-mer sequence were used for prediction with the MT-CNN model and the taxonomic and genomic region predictions for each window were ensembled to select the most likely overall taxonomic and genomic region label using a hard-voting strategy: predictions of ≥ 0.9 were allocated a vote, and the taxonomic and genomic region label with the most votes were assigned to the overall input read.

### Taxonomic ranking using a naive Bayes approach

A naïve Bayes approach was used for taxonomic ranking of candidate viruses from the assigned reads and genomic coverage of reads derived from the MT-CNN model. From the MT-CNN outputs, the number of reads per genomic region (1–10) assigned to the same viral species were tabulated prior to estimation of the genomic coverage for each species. To account for errors in genome region assignments, only regions that met a pre-defined threshold were counted, which was set at 0.1 of the average number of expected hits per genomic region, assuming a uniform distribution:

1 $$ {\text{Threshold  =  (total}}\;{\text{number}}\;{\text{of}}\;{\text{ hits}}\;{\text{for}}\;{\text{all}}\;{\text{species)}}\; \times \;{\text{0}}{\text{.1/(total}}\;{\text{ number}}\;{\text{ of}}\;{\text{ covered}}\;{\text{ regions)}} $$

For the implementation of the naïve Bayes network, the number of assigned reads and the number of genomic regions per species were discretized (Additional file 1: table S1) for inputs as parent nodes. Joint probabilities in conditional probability tables (CPTs) of the naive Bayesian network for calculating the likelihood estimate of a taxonomic species label were generated [17] based on relative weights of influence by the number of assigned reads and coverage of assigned reads, set at 7:3. The network was implemented using a Python library pgmpy 0.1.10 [18] (https://pgmpy.org/).

### Generation of training/testing datasets

The datasets for training/testing were derived from the International Committee on Taxonomy of Viruses (ICTV) (https://talk.ictvonline.org/) database that provides high-quality genomes of viruses with the standard taxonomy labels. To obtain only human viruses from ICTV, 434 human viral genomes from 187 species were extracted from ICTV by cross referencing them to ViralZone [19], a database that maintains a list of human viruses and their accession IDs.

For preprocessing of sequences prior to training the MT-CNN model, the viral genomes were split into ~ 10 million unique 50-mer sequences and then augmented using Mason2 (https://github.com/seqan/seqan/tree/master/apps/mason2) to generate additional datasets with different mismatch, insertion, and deletion rates (Additional file 1: table S2) in order to improve the generalizability of predictions. In total, the merged datasets comprised ~ 53 million unique *k*-mers. These *k*-mers were then split as training/validation/testing datasets at a ratio of 51:1:1 and the bulk of sequences (~ 51 million unique 50-mers) were used for training the MT-CNN model.

For baseline evaluation of the MT-CNN model, the 50-mer testing dataset of 1,000,000 sequences was used, while a 150-mer dataset of 100,000 sequences was generated from the 434 human viral genomes of ICTV for the evaluation of longer sequences. For all datasets, the species labels and the genomic region labels were used for evaluating the performance of the model on taxonomic assignment and prediction of genomic regions respectively.

### Simulated datasets of divergent genomes (HIV-1)

For evaluation of performance on divergent sequences, divergent viral genomes of HIV-1 were generated using SANTA-SIM [20], which simulates viral sequence evolution dynamics under selection and recombination. Using the default settings (https://github.com/santa-dev/santa-sim/blob/master/examples/HIV-1.xml), three different HIV-1 datasets were simulated with different number of generations to reflect divergence as the virus evolves. Each dataset contained 1,000 simulated HIV-1 genomes after 100, 1000 and 10,000 generations, representing ~ 2–3 months, 1–3 years and 10–30 years of infection respectively (assuming a replication cycle of 1–2 days). Each HIV-1 genome dataset was then used to generate 100,000 reads of 150 bp in length using Mason2.

### Real-world datasets (SARS-CoV-2)

Four recently published clinical RNAseq datasets of SARS-CoV-2 viruses from the University of Washington were used for evaluation of performance on real-world data [21] on a species that was not present in the training datasets. The RNAseq datasets had a maximum sequence length of 185 bp and they were filtered for reads shorter than 50 bp (0.55%) prior to analysis.

### Benchmarking tools and metrics

For testing the performance of our MT-CNN model on the taxonomic assignment task, several state-of-the-art tools were chosen for benchmarking: Kraken 2, Centrifuge and Bowtie 2 [22]. These tools were installed with their default databases (MiniKraken2_v2_8GB database for Kraken 2 https://ccb.jhu.edu/software/kraken2/index.shtml?t=downloads, and compressed human, bacteria, archaea and viruses database for Centrifuge https://genome-idx.s3.amazonaws.com/centrifuge/p_compressed%2Bh%2Bv.tar.gz). For Bowtie 2, human viral genomes from ICTV (n = 434) were used to build the reference database.

To evaluate the performance of taxonomic assignments, the following metrics were adopted for the different types of datasets: (1) for the simulated NGS reads from standard genomes of ICTV, Cohen’s kappa coefficient which measures inter-rater reliability for categorical items, and F1 score which is the harmonic mean of the precision and recall, were used as the metrics for multi-class prediction; (2) for the simulated divergent reads from HIV-1 datasets, prediction accuracy which represents the percentage of correctly identified reads in all reads was used; (3) for the real-world RNA-Seq datasets, the sensitivity of classification of SARS (the closest relation with SARS-CoV-2) was used.

To evaluate the performance of genomic position assignments, Cohen’s kappa coefficient and F1 score were adopted as the metrics for multi-class prediction of the genomic position of taxonomically labelled reads for the MT-CNN model and Bowtie2, as both Kraken and Centrifuge did not provide any positional assignments.

All performance evaluations were implemented and calculated using the scikit-learn library version 0.21.2 (https://scikit-learn.org/stable/).

## Results

As part of a pipeline to provide a more robust identification of human viruses that may be divergent (Fig. 2), we first developed a multi-task CNN (MT-CNN) model that combined 2 tasks of assigning the taxonomic label and its genomic position into a single end-to-end deep learning model (Fig. 3). As the deep learning model takes a fixed-length input, we chose a 50-mer length sequence that would balance the model’s ability to learn features (Additional file 1: figure S1) with the model complexity and flexibility of analyzing longer sequences using a sliding window strategy.

To optimize and train the model, we generated a human viral dataset derived from ICTV comprising 187 species of 434 reference genomes, augmented with different mutations to increase generalizability of predictions by the deep learning model. The initial model was tuned to optimize prediction of both tasks on human virus dataset. Using optimized parameters—convolutional kernels (5 and 7), dropout rate (0.2) and momentum of the batch normalization layers (0.6) – we trained the final MT-CNN model on the larger augmented dataset of 51 million 50-mer sequences with an early-stopping strategy on the validation dataset to reduce overfitting.

To evaluate the performance of the MT-CNN model on both the taxonomic and the genome region assignment tasks, we first compared our MT-CNN model to state-of-the-art alignment-based and alignment-free tools using the baseline 50-mer and a longer 150-mer dataset.

### Evaluation of baseline taxonomic assignment performance on test datasets from ICTV human viral genomes

To evaluate the baseline performance of the MT-CNN model for taxonomic assignment, we first used the 50-mer test dataset of 1,000,000 sequences separate from the training dataset, both split from the original dataset derived from 434 ICTV human viral genomes. Because of the multi-class prediction of 187 species labels, we used 2 metrics for evaluation: Cohen’s kappa coefficient which measures inter-rater reliability for categorical items and the F1 score, the harmonic mean of the precision and recall. We found that on the simulated 50-mer dataset, the MT-CNN model (Kappa = 0.9464, F1 = 0.9475) outperformed Kraken 2 (Kappa = 0.3139, F1 = 0.3184) and Centrifuge (Kappa = 0.5953, F1 = 0.6011), and was comparable with Bowtie 2 (Kappa = 0.9597, F1 = 0.9605) (Table 1).

**Table 1:** Benchmarking of taxonomic assignment tasks using simulated reads (ICTV and HIV-1)

Datasets	Simulated reads (50 bp, n = 1million) from ICTV standard genomes		Simulated reads (150 bp, n = 100 k) from ICTV standard genomes		Simulated divergent HIV-1 reads (150 bp, n = 100 k) (100 generations)	Simulated divergent HIV-1 reads (150 bp, n = 100 k) (1 K generations)	Simulated divergent HIV-1 reads (150 bp, n = 100 k) (10 K generations)
Metrics	Kappa	F1	Kappa	F1	Prediction accuracy	Prediction accuracy	Prediction accuracy
Kraken 2	0.3139	0.3184	0.7274	0.7318	0.9664	0.7755	4.890 × 10^–3^
Centrifuge	0.5953	0.6011	0.7219	0.7266	0.8865	0.8679	3.056 × 10^–2^
Bowtie 2	0.9597	0.9605	0.9991	0.9991	0.9976	0.9828	8.501 × 10^–4^
MT-CNN	0.9464	0.9475	0.9976	0.9976	1.000	0.9963	0.1531

To evaluate the baseline performance of the MT-CNN model on longer reads typically used in short-read sequencing, we incorporated a sliding window strategy that produces an ensembled prediction of a longer sequencing read from 50-mer window predictions generated by the MT-CNN model. Using a simulated 150-mer test dataset of 100,000 sequences, we found that the performance of our MT-CNN model (Kappa = 0.9976, F1 = 0.9976) improved significantly and still outperformed Kraken 2 (Kappa = 0.7274, F1 = 0.7318) and Centrifuge (Kappa = 0.7219, F1 = 0.7266), and maintained a comparable performance to Bowtie 2 (Kappa = 0.9991, F1 = 0.9991) (Table 1).

### Evaluation of baseline genome region assignment performance on test datasets from ICTV human viral genomes

Next, we evaluated the performance of the MT-CNN model on genome region assignment of sequences using the simulated 50-mer and 150-mer datasets derived from 434 ICTV human viral genomes. Given the multi-class prediction of genomic regions, we used Cohen’s kappa coefficient and the F1 score to evaluate the performance of the predictions from the MT-CNN model compared to the Bowtie2 aligner; both Kraken 2 and Centrifuge were not included as they do not provide any genome position/region assignments for evaluation. We found that our MT-CNN model performed well (Kappa = 0.9336–0.9405, F1 = 0.9403–0.9465) on genome region assignment at comparable levels to Bowtie2 (Kappa = 0.9442–0.9646, F1 = 0.9498–0.9646) for both 50-mer and 150-mer datasets (Table 2), indicating that the predictions were sufficiently accurate for downstream analysis in the pipeline.

**Table 2:** Benchmarking of genomic coverage tasks using simulated reads (ICTV)

Datasets	Simulated reads (50 bp, n = 1million) from ICTV human viral genomes		Simulated reads (150 bp, n = 100 k) from ICTV human viral genomes	
Metrics	Kappa	F1	Kappa	F1
Bowtie 2	0.9442	0.9498	0.9646	0.9646
MT-CNN	0.9336	0.9403	0.9405	0.9465

### Evaluation of taxonomic assignment performance on divergent sequences using simulated evolution of HIV-1 genomes

Having established the baseline performance of the MT-CNN model on taxonomic and genome position assignments, we next wanted to test if the model had learnt features that conferred sensitivity in identifying human viral sequences that were highly divergent. To do this, we simulated 150-mer divergent sequences based on the HIV-1 genome using SANTA-SIM [20], which takes into account evolutionary dynamics as a viral genome evolves from generation to generation. Using this tool, we generated datasets of divergent HIV-1 sequences after 100, 1000 and 10,000 generations.

To evaluate the ability of our model to detect divergent sequences from the original HIV-1 genome, we considered the prediction accuracy as the percentage of correctly identified reads from the total reads (Table 1). We found our MT-CNN model consistently outperformed the other methods in detecting HIV-1 sequences in the 3 datasets representing divergent sequences after 100, 1000, and 10,000 generations. The sensitivity of detecting HIV-1 was higher for more divergent sequences; notably after 10,000 generations, the MT-CNN model was 5x (Centrifuge) to 180x (Bowtie2) more sensitive at identifying HIV-1 even though the overall performance decreased significantly compared to the less divergent datasets (100 and 1000 generations) indicating that there is scope for further optimization in learning features in our model.

### Evaluation of taxonomic assignment performance on real-world datasets representing novel and emerging viral genomes

To evaluate the generalizability of the MT-CNN model predictions on real-world datasets representing novel and emerging viruses, we used four recently published clinical RNAseq datasets of SARS-CoV-2 viruses [21] that were not present in the training dataset for our model nor in the reference datasets for the other tools.

We first subtracted all human sequences from the data and aligned the remaining reads (maximum length 185 bp) using BLAST against the NCBI nt database prior to the emergence of SARS-CoV-2 (190 GB, released in Sep-3rd-2018) to determine the closest related virus that could be identified. Of the aligned reads, most (~ 52%) were identified as SARS, the closest relation of SARS-CoV-2. We therefore used the sensitivity of SARS classification as a metric for evaluating the ability of our model and the other tools to identify the closest relation of SARS-CoV-2.

**Fig. 5:**
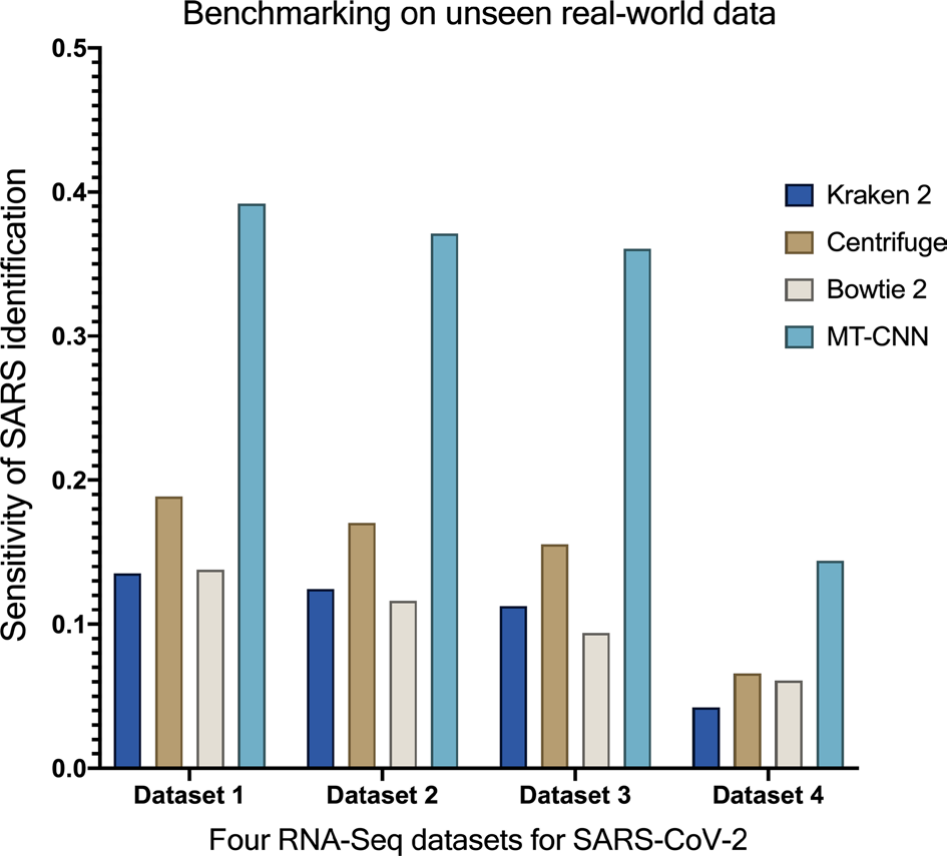
Benchmarking of virus identification accuracy in an out-of-sample real-world dataset. Four RNAseq files of SARS-CoV-2 [21] were analysed by benchmarking tools to determine the sensitivity of classified SARS-related viral reads as the closest relation to SARS-CoV-2

We found that the MT-CNN model consistently achieved the highest sensitivity of SARS classification, the closest relation to SARS-CoV-2, from all 4 datasets as compared to the other tools (Fig. 5). Consistent with our finding of decreased sensitivity in highly divergent HIV-1 simulated sequences, the low sensitivity of classification in some datasets likely reflects the highly divergent nature of SARS-CoV-2 from the reference sequences used from comparison.

Interestingly, although a low number of reads were identified in RNAseq dataset 4 (Table 3), the reads assigned to SARS were the highest among the other candidate viruses, and importantly, the reads were broadly distributed by genomic region providing greater support that the sample likely contains a virus that is related to SARS.

**Table 3:**
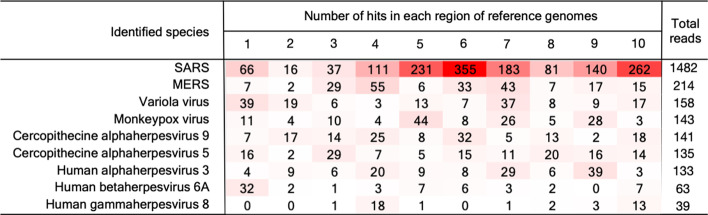
The output report of the MT-CNN model

The number of hits per identified species and the predicted genomic region for the sample sequence data were generated from SARS-CoV-2 dataset 4

**Table 4:** A taxonomic report of the MT-CNN/Bayesian pipeline

Identified species	Percentage of assingned hits	Number of covered regions	RanK based on percentage	RanK based on Bayesian scores
SARS	0.5969	10	1	1
MERS	0.0854	9	2	2
variola virus	0.0624	9	3	2
Cercopithecine alphaherpes virus 9	0.0560	9	4	2
Cercopithecine betaherpes virus 5	0.0536	9	5	2
Monkeypox virus	0.0532	7	6	7
Human alphaherpes virus 3	0.0507	8	7	6
Human alphaherpes virus 6A	0.0209	4	8	8
Human mastadenovius G	0.0125	1	9	17
Human gammaherpesvirus 8	0.0125	2	9	11

The table shows the output from the pipeline using the SARS-CoV-2 dataset 4. The initial ranking was done based only of the percentage of assigned hits and the Bayesian ranking incorporated information about the genomic coverage

### Taxonomic ranking using a naïve Bayesian network for integrating taxonomic and genomic region assignments

Given that both taxonomic assignment of reads and the genomic coverage of assigned reads can influence the confidence of identification of potential viral pathogens, we were motivated to develop a ranking approach that integrates both dimensions as part of the pipeline. To do this, we implemented a naïve Bayesian network that takes into account the proportion of reads assigned to a species and the degree of coverage of genomic regions, both derived from downstream outputs from the MT-CNN model. In this network, both taxonomic assignments and degree of coverage of genomic regions were discretized and used to build a conditional probability table based on the relative weightage of importance assigned to both parameters.

To illustrate the taxonomic ranking approach, we used the SARS-CoV-2 RNAseq dataset 4 to generate a ranking list of potential viruses in the sample based solely on the percentage of assigned reads to each species (Table 4). Using the Bayesian network, we updated the ranks by incorporating information about the coverage of genomic regions of each species (Table 4). We found that the ranking of the viral species changed in accordance of the assigned reads and the coverage; for example, the rank of the Monkeypox virus dropped from 6 to 7 due to the low genomic coverage despite having more assigned reads, while the human alphaherpesvirus 3 was re-ranked from 7 to 6 given its higher genomic coverage although the assigned reads were fewer. The intuitive changes in ranking by incorporating prior information suggests that this approach could be extended using other parameters that could be helpful for the interpretability of a pathogen report where multiple candidate viruses have been identified.

## Discussion

In this paper, we describe the development of a pipeline that incorporates a multi-task convolutional neural network model (MT-CNN) with a Bayesian ranking approach to identify and rank the most likely human virus from samples containing divergent reads. We evaluated the baseline characteristics of the MT-CNN model after training it on ICTV human viruses and showed that on the baseline 50-mer and 150-mer test datasets, our model was able to outperform or match the performance of other state-of-the-art tools, Kraken 2, Centrifuge and Bowtie 2, indicating the network had learnt features for performant classification of 187 human viruses. Similarly, the performance of our novel multi-task architecture that allows for the simultaneous assignment of genomic regions showed metrics comparable to the Bowtie 2 aligner.

Deep learning approaches, including our MT-CNN model, have the potential for learning features from sequence data for classification of divergent sequences, particularly from novel and emergent viruses. Consistent with this idea, we evaluated the MT-CNN model on divergent sequences from simulated evolution of HIV-1 genomes after 100 to 10,000 generations and showed that the model outperformed the other tools in detecting HIV-1 sequences and the sensitivity gap was higher as the sequence divergence increased. This observation was also consistent when our model was tested on SARS-CoV-2 datasets that were not in the training data or in the reference databases; the MT-CNN model outperformed the other tools in detection of SARS, the closest relation to SARS-CoV-2, indicating that it may potentially be useful in the detection of novel and emergent viruses in the future. Interestingly, we noted that although the model’s sensitivity was higher than the other tools, the overall sensitivity for detecting divergent HIV-1 or SARS-CoV-2-related sequences was relatively low, suggesting additional opportunities exist for optimization and use of other architectures to improve the performance of the model.

Another important feature of our pipeline is the incorporation of a naïve Bayes network for ranking of taxonomic assignments by considering both the number of read assignments and the genomic coverage per identified species generated by the MT-CNN model. We showed that our implementation was able to re-rank taxonomic assignments according to the weightage of importance assigned to the number of reads and the genomic coverage of the assigned species. The network could potentially be extended to include other relevant data that could increase the confidence of a taxonomic assignment where several candidates have been identified.

## Conclusions

In summary, we have developed a pipeline that combines a novel MT-CNN model that is able to identify viruses with divergent sequences together with assignment of the genomic region, with a Bayesian approach to ranking of taxonomic assignments by taking into account both the number of assigned reads and genomic coverage. We envision that further developments in the architecture for improving the accuracy and the inclusion of other relevant clinical data into the Bayesian network, would enable an integrated taxonomic report that is accurate and interpretable for clinical use in the identification of human viral pathogens.

## Supplementary Information

**Additional file 1.** **Figure S1**: Testing the learning ability of MT CNN using differentlengths of k mers. **Table S1**: Transformation of percentage of assigned reads to discretevariables for the naive Bayesian network. **Table S2**: The insertion, deletion, and mismatch rates for simulating50 mers using Mason2.

## Data Availability

The pre-trained MT-CNN model and related scripts are available on GitHub via https://github.com/MaHaoran627/CNN_Virus. The training/validation datasets and simulated HIV-1 genomes are available via Google Drive (https://drive.google.com/open?id=1sj0-NCSKjLta_Geg6EMo26rChmtWcOiI). The SARS-CoV-2 datasets [21] were obtained from NCBI SRA under the accession IDs SRX7852917, SRX7852918, SRX7857604, SRX7857605.

## Abbreviations

MT-CNN: Multi-task convolutional neural network
ReLU: Rectified linear unit
ICTV: International Committee on Taxonomy of Viruses
CPT: Conditional probability table
NGS: Next generation sequencing
