# Supplementary material for: A multi-task CNN learning model for taxonomic assignment of human viruses
Source: BMC Bioinformatics. 2021 Jun 2;22(Suppl 6):194. doi: 10.1186/s12859-021-04084-w (PMC8170063; doi:10.1186/s12859-021-04084-w)
Supplement: Supplementary file 1 — Additional file 1. Figure S1: Testing the learning ability of MT CNN using differentlengths of k mers. Table S1: Transformation of percentage of assigned reads to discretevariables for the naive Bayesian network. Table S2: The insertion, deletion, and mismatch rates for simulating50 mers using Mason2. [file 12859_2021_4084_MOESM1_ESM.docx]

**Additional file 1 of A Multi-task CNN Learning Model for Taxonomic Assignment of Human Viruses**


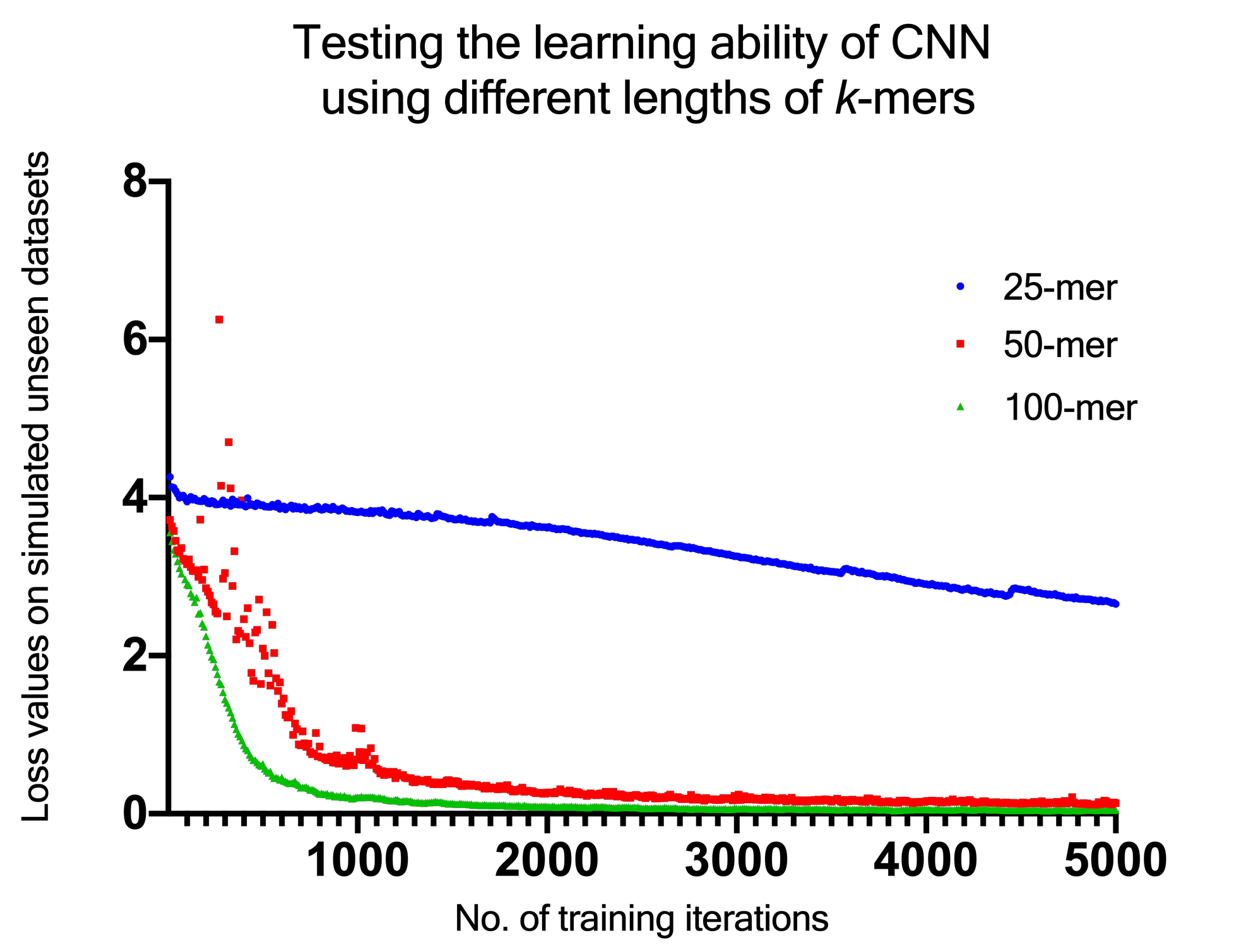


**Additional file 1 figure S1. Testing the learning ability of MT-CNN using different lengths of k-mers.** Three datasets were generated from 434 human viral genomes in ICTV as 25-mers, 50-mers and 100-mers, respectively. The datasets were split into training and test datasets. Three CNN models were trained using the same architecture and parameters for 5000 iterations (batch size equals to 512). The loss values on test datasets were recorded.

**Additional file 1 table S1. Transformation of percentage of assigned reads to discrete variables ^a^ for the naive Bayesian network.**

| The percentage of assigned reads | Transformed discrete variables |
| --- | --- |
| <1% | 0 |
| [1%, 3%) | 1 |
| [3%, 5%) | 2 |
| [5%, 10%) | 3 |
| [10%, 20%) | 4 |
| [20%, 30%) | 5 |
| [30%, 50%) | 6 |
| >=50% | 7 |

**^a^** The percentage of assigned reads was transformed to 8 discrete variables.

**Additional file 1 table S2. The insertion, deletion, and mismatch rates for simulating 50-mers using Mason2.**

|  | **Insertion rate** | **Deletion rate** | **Mismatch rate** |
| --- | --- | --- | --- |
| **Dataset 1** | 0.001 | 0.001 | 0.004 |
| **Dataset 2** | 0.00125 | 0.00125 | 0.005 |
| **Dataset 3** | 0.0015 | 0.0015 | 0.006 |
| **Dataset 4** | 0.00175 | 0.00175 | 0.007 |
| **Dataset 5** | 0.002 | 0.002 | 0.008 |
| **Dataset 6** | 0.00225 | 0.00225 | 0.009 |
| **Dataset 7** | 0.0025 | 0.0025 | 0.01 |
| **Dataset 8** | 0.00275 | 0.00275 | 0.011 |
| **Dataset 9** | 0.003 | 0.003 | 0.012 |
